# The Evolving Role of Radiosurgery in the Management of Radiation-Induced Meningiomas: A Review of Current Advances and Future Directions

**DOI:** 10.1155/2014/107526

**Published:** 2014-07-17

**Authors:** Alireza Mansouri, Jetan Badhiwala, Sheila Mansouri, Gelareh Zadeh

**Affiliations:** ^1^Division of Neurosurgery, University of Toronto, Toronto, Canada M5T 2S8; ^2^Department of Neurosurgery, Toronto Western Hospital, University Health Network, 4W-436, 399 Bathurst Street, Toronto, ON, Canada M5T 2S8

## Abstract

Meningiomas are among the most common primary adult brain tumors, which arise either spontaneously or secondary to environmental factors such as ionizing radiation. The latter are referred to as radiation-induced meningiomas (RIMs) which, while much less common than their spontaneous counterparts, are challenging from a management point of view. Similar to spontaneous meningiomas, the optimal management of RIMs is complete surgical resection. However, given their high grade, multiplicity, tendency to invade bone and venous sinuses, and high recurrence rate, this cannot always be accomplished safely. Therefore, other therapeutic modalities, such as stereotactic radiosurgery, have emerged. In the current review, we provide an overview of the historical outcomes achieved for RIMs through radiosurgery and microsurgical resection. Furthermore, we provide a discussion of clinical and radiological parameters that affect the decision-making process with regard to the management of RIMs. We also provide an outline of recent changes in our understanding of RIMs, based on molecular and genetic markers, and how these will change our management perspective. We conclude the review by summarizing some of the current obstacles in the management of RIMs with SRS and how current and future research can address these challenges.

## 1. Introduction

Meningiomas are the second most common adult brain tumour, typically presenting in the 5th-6th decade of life [1]. Meningiomas can arise spontaneously secondary to various genetic alterations with mutations on chromosome 22 being the most common [2]. In addition to genetic alterations, however, environmental factors such as exposure to ionizing radiation have been implicated in their pathophysiology as well. In a large study in 1988, Ron and colleagues were the first to show that patients exposed to ionizing radiation at a young age for treatment of tinea capitis were at a 9.5-fold higher risk of developing intracranial meningiomas—thus called radiation-induced meningiomas (RIMs)—within the field of radiation [3]. Later in 1999, Shintani et al. were able to show that survivors of the Hiroshima and Nagasaki atomic bomb explosions were also at a significantly higher risk of developing meningiomas [4].

In comparison to spontaneous meningiomas (SMs), patients with RIMs are typically of a younger age at diagnosis, tend to present with multiple lesions, and demonstrate a higher recurrence rate [5, 6]. Histopathological analyses have suggested that RIMs are also more aggressive than their spontaneous counterparts [7, 8]. A number of genetic aberrations have been detected in these lesions, including chromosomes 1p, 6p, 6q, 7p, and 22q [7–10]. The mean latency from irradiation to tumor appearance is approximately 38 years, ranging from 2 to 63 years [3, 11]. Some authors have observed a shorter latency period in patients receiving radiation prior to the age of 5 years [11, 12]. This implies that immature nervous tissue is more susceptible to chromosomal injury following irradiation and that tumorigenesis in RIMs occurs following a long quiescent phase.

The available evidence suggests that RIMs differ biologically and clinically from SMs. Likewise the management of RIMs presents unique challenges and merits special discussion. In this review, we will provide a brief overview of outcomes currently achieved through various modalities utilized in the management of RIMs with a focus on the role of radiation therapy (RT) and predominantly in the form of stereotactic radiosurgery (SRS) and how these compare with SRS outcomes in SMs and microsurgical outcomes with RIMs. Subsequently, we will provide a discussion on clinical and radiological factors that must be considered as part of the decision-making process in the management of RIMs. Further, we will describe some of the molecular and genetic discoveries that have transformed our understanding of these lesions. We conclude by exploring some of the challenges associated with the use of SRS in the management of RIMs and how current and future research may address some of these questions.

## 2. Current Therapeutic Modalities for RIMs

Similar to SMs, the first approach in the management of RIMs has typically been complete surgical resection, including resection of the associated dura and bone (Simpson Grade I) [13]. However, the aggressive growth nature of RIMs and their multiplicity often hinders a gross surgical resection, necessitating other treatment modalities. Paradoxically, radiation-based modalities such as SRS can be used. Recent studies have shown that adjuvant or primary SRS is indeed a safe and efficacious modality in the management of RIMs (Table 1) [6, 14, 15]. Jensen and colleagues, assessing 16 patients with 20 radiation-induced tumors (19 of them meningiomas) treated with SRS as the primary modality, were able to demonstrate a 5-year local control and overall survival (OS) rate of 100% and 80%, respectively. The median follow-up in this study was 40.2 months and the median marginal dose was 16 Gy (range 12–20 Gy) [14]. In a study of 19 patients with 24 total RIMs, Kondziolka and colleagues showed a 75% tumor control rate and a combined morbidity rate of 5.3% during a median follow-up of 44 months when SRS was used as primary or adjuvant therapy. In this cohort, an OS rate of 100%, 94.1%, and 80.7% was demonstrated at 1, 3, and 5 years, respectively [6]. The progression-free survival (PFS) rate after SRS was 95.8%, 78.9%, and 66.2% at 1, 3, and 5 years, respectively. A marginal dose of 13 Gy or greater was found to be a significant predictor of improved PFS. Similar to SMs, tumor size was an important predictor of success. In tumors with volume ≤5 mL, only 1 of 14 progressed at 81.4 months, whereas 5 of 10 patients with volumes >5 mL demonstrated local progression with a median time to progression of 42.2 months. Tumors ≤5 mL had a PFS rate of 100%, 88.9%, and 88.9% at 1, 3, and 5 years; whereas, those greater than 5 mL had a PFS rate of 90%, 65.6%, and 35% at 1, 3, and 5 years.

The tumor control rate seen with RIMs is roughly similar to that reported for SMs treated with SRS. Depending on the grade and location of the lesion, the reported 5-year tumor control rates of SMs with SRS have ranged from 86 to 99% [16–20]. The results are less favorable for higher grade lesions with studies demonstrating 2- to 5-year control rates of less than 50% in Grade II and 0–17% for Grade III lesions, respectively [20–28]. Therefore, although it has been suggested that RIMs are typically more aggressive in nature than SMs, the tumor control rate appears to be in between that of Grade I and Grade II/III SMs.

The recurrence rate of RIMs managed solely through surgical resection has ranged from 0 to 43%, depending on the length of follow-up and whether radiotherapy (RT) was used concurrently (Table 2) [5, 30–33]. De Tommasi and associates reported six operated cases of RIMs [31]. Total tumor removal was achieved in all cases. The reported recurrence rate was 0%. On the other end of the spectrum, Harrison et al. (1991) reported a recurrence rate of 43% among seven patients undergoing resection of RIMs [5]. One explanation for this discrepancy in recurrence rate relates to the amount of radiation adminsitered. All patients in De Tommasi and colleagues' series had received low-dose cranial irradiation for treatment of tinea capitis. On the other hand, Harrison and colleagues included patients treated by low-, moderate-, and high-dose cranial RT for various indications. Two of the patients who had recurrences had been treated with high-dose irradiation for brain tumors. Meningiomas arising from high-dose RT tend to be malignant, whereas those arising from low-dose RT are usually benign [29, 30]. This obviously has important implications for local tumor control. Indeed, 3 patients (nearly 50%) treated by Harrison and group had atypical meningiomas, whereas only one patient had a high-grade meningioma in De Tommasi et al.'s cohort. The largest surgical series of RIMs comes from Sadetzki et al. in Israel [30]. This group examined a cohort of patients who were treated with ionizing radiation in childhood for tinea capitis. Among 220 patients with RIMs treated by resection, the recurrence rate was 18.2%, which was not significantly different from a control group of patients with SMs (14.6%). A recurrence rate on the order of 20% appears to be a fair estimate based on a number of different studies [34, 35].

Although complete surgical resection, to the safest extent possible, has traditionally been the first line approach for both RIMs and SMs, the improvement of radiosurgical instruments has shifted the management paradigm to include both modalities as required. In the following section, we will review and critically analyze factors that should be considered in deciding upon one management modality over another or in combinations, as deemed necessary.

## 3. Selecting the Optimal Management Modality

As with other pathological entities in medicine, an accurate history and physical examination is the cornerstone of the decision-making process in the management of RIMs as well. Patients with RIMs are typically divided into those with histories of low, medium, and high dose radiation [5]. As discussed briefly above, some suggest that patients with a prior history of high dose radiation are more likely to present with higher grade lesions and have a greater likelihood of recurrence [3–5, 11, 29, 30, 34, 36–38]. Therefore, following surgical resection, this cohort of patients is more likely to require adjuvant therapy particularly if a Simpson I resection has not been obtained. Furthermore, closer clinical and radiological follow-up is required as well to monitor for recurrence. In these patients, accurate documentation of prior treatments and radiation dosing (if radiation has been used as part of treatment) is particularly essential given that a previous history of radiation can result in a higher risk of adverse outcomes in subsequent radiation sessions [26]. While some authors offer SRS only when maximal RT through other modalities has been exhausted [36], others do not use this variable as a deciding factor [6, 39].

The age at presentation is important as well. While multiplicity is common with RIMs, patients presenting at a younger age have been found to be at a greater predisposition [6, 40]. The decision to treat certain lesion(s) over another and the optimal treatment modality can be challenging. This decision would depend largely on clinical and radiological parameters.

Clinically, it is important to correlate the lesion(s) with symptom presentation and to assess the risk of surgical intervention. In patients in whom a gross total resection (GTR) may be a high risk endeavor, it may be prudent to proceed with a planned subtotal resection (STR) followed by adjuvant therapy. The overall status of the patient is important as well. In particular, but not limited to, the status of the patient scalp is essential: given the history of prior radiation, the scalp of some patients is thin and prone to necrosis, dehiscence, and CSF leakage, increasing the overall risk of complications of surgical intervention [36]. Ultimately, the patient as a whole must be considered in order to decide upon proceeding with surgery and whether additional measures such as a cranioplasty or skin grafting would be required. In some of these patients, SRS may be a safer option.

Radiological parameters that can be used to identify the lesion(s) that are more actively growing include the degree of T2 hyperintensity within the lesion(s), surrounding edema, absence of calcification (as seen on CT), or evidence of rapid enlargement on serial imaging [41, 42]. In addition to larger lesion size and location, the presence of peritumoral edema and partial/complete occlusion of venous sinuses has been associated with a greater likelihood of adverse outcomes following SRS for SMs as well. Based on these principles surgical resection, to the safest extent possible, may be a better option for RIMs with such features [43, 44].

The invasion of major venous sinuses by RIMs and their multiplicity is a major obstacle to intervention, whether in the form of surgical resection or SRS. However, advances in SRS technique that have been applied in the management of challenging SMs offer hope. Recently, Deibert and Kondziolka have reported the case of a patient presenting with a large recurrent meningioma invading the posterior two-thirds of the superior sagittal sinus, extending 16 cm in its greatest length [45]. This patient had previously undergone three separate interventions (surgical resection, gamma knife-based SRS, and intensity-modulated RT). Through a division of the lesion into three side-by-side dose matrices and applying a marginal and maximal SRS dose of 12 Gy and 24 Gy, respectively, the patient has experienced tumor regression and has been recurrence-free over a 5-year period. Reporting on a cohort of 73 patients with multiple SMs (primary or recurrent, 221 total lesions) managed with linear accelerator-based SRS, Samblas and colleagues have demonstrated a 90% tumor control rate over a median follow-up of 5.3 years [46]. While these advances are encouraging, they apply to SMs and represent individual studies only. Further studies are necessary to confirm and elaborate on these findings and determine their applicability to RIMs. In addition, other concerns such as the management of cases with extensive bony involvement need to be addressed as well: RIMs have a high propensity toward calvarial involvement, which increases the risk associated with surgical resection and decreases the likelihood of success with SRS/RT [6, 34].

## 4. Molecular and Genetic Markers

Traditionally, meningiomas have been grouped based on the most recent WHO classification [47]. However, the discovery of recent molecular and genetic markers may allow for better stratification of these lesions and improve our management strategies. While RIMs have a greater proportion of high grade lesions compared with SMs, recurrence has been observed in some WHO Grade I RIMs as well [8, 11, 31, 48]. Several authors have demonstrated that a higher MIB-1 index is associated with lesion multiplicity and recurrence in patients with RIMs [8, 11, 49–51]. Interestingly, in a small study of pediatric patients with RIMs, the MIB-1 index has been shown to be similar to patients with SMs and the authors have suggested that other cytogenetic aberrations are involved [7]. Pistolesi and colleagues, examining the case of an adult patient with RIM and multiple recurrences and metastases, demonstrated an increased expression of p53 and increased telomerase activity [52]. Although benign meningiomas have an overall lower telomerase activity, patients with Grade I meningiomas with an increased level of activity are at a greater risk of tumor recurrence even after GTR and, therefore, are more likely to require postoperative adjuvant radiation [53, 54]. Patients with high grade RIMs or those with multiple recurrences have been shown to have an increased vascularity and high levels of vascular endothelial growth factor (VEGF) mRNA expression as well; the expression of VEGF increases the risk of peritumoral edema, particularly following SRS [52, 55, 56]. These findings demonstrate that the accurate prediction of the natural history and response of RIMs to treatment is beyond the criteria assessed through the traditional WHO classification. The incorporation of molecular and genetic markers will undoubtedly help tailor the management algorithm to each individual patient.

## 5. Future Directions

Although RIMs are relatively uncommon, they represent a challenge from a therapeutic perspective. In addition, the increasing survival of patients with childhood malignancies implies that the prevalence of RIMs is likely to increase in the near future. The fact that there is a long delay in occurrence of RIMS suggests that there is memory of radiation injury or long-term molecular alterations that result in RIM formation. Therefore, understanding the molecular alterations induced by radiation therapy is critical in the prevention of RIM formation. This is a disease model that lends itself ideally to research, and understanding of its molecular pathophysiology can provide insights and a better understanding of meningiomas in general with ultimate therapeutic targets being identified.

The role of adjunctive medical therapies is also an area for further investigation. To date, several agents—such as hydroxyurea—have been attempted with variable success rates [36]. Recently, Bevacizumab—a monoclonal antibody targeting VEGF—has been utilized in the management of SMs presenting with edema—in isolation or following SRS—and for the prevention of recurrence with reasonable success [57, 58]. Lou et al. have utilized Bevacizumab in the management of 14 patients with recurrent SMs in whom multiple prior resections and external beam RT had been attempted, demonstrating a 6-month PFS of 86% [58]. Puchner et al. have reported the use of Bevacizumab in the case of a patient with a recurrent anaplastic meningioma—following surgical resection and RT—demonstrating a durable regression over a 6-month follow-up period [59]. The long-term success of Bevacizumab and its applicability to the management of RIMs remain to be determined.

With an increased understanding of RIMs and an expansion of the available therapeutic armamentarium, clinicians may be able to gradually push the envelope when it comes to the multimodal management of RIMs. SRS has been shown to represent a safe and efficacious option in the management of RIMs implying that radical surgical resection may be gradually replaced by a planned STR that is safe, followed by adjuvant SRS therapy. The detection of markers that predict a greater likelihood of recurrence will also help decide upon the optimal follow-up routine and the need for adjuvant therapy. The role of therapeutic drug agents targeting aberrant molecular pathways is paramount as well. While many obstacles remain when it comes to the management of RIMs, great strides have been made. It is essential to learn from past experiences while remaining open to the integration of new treatment strategies and targets.

## Figures and Tables

**Table 1:** Series of radiation-induced meningiomas treated by stereotactic radiosurgery.

Author and year	*N*	Original diagnosis for RT	Mean age (yrs.)∗	F/M	Mean RT dose (Gy)	Mean latency (yrs.)	Mean SRS dose (Gy)	Mean tumor volume (cm^3^)	WHO grade	Mean follow-up (yrs.)	Morbidity	Tumor progression
Jensen et al., 2005 [14]	16	3, pituitary tumor 3, astrocytoma 2, tumor NOS 1, oligodendroglioma 1, tinea capitis 1, craniopharyngioma 1, neuroblastoma 1, ALL 1, glioma 1, esthesioneuroblastoma 1, spindle cell tumor	47.5	10/6	55.6 (*n* = 12)	32.3	margin, 15.9	10.0	—	3.4	1 (6.3%) −1, increased seizure activity	1/19 (5.3%)

Kondziolka et al., 2009 [6]	19	4, pituitary adenoma 3, medulloblastoma 2, leukemia 2, astrocytoma 1, pilocytic astrocytoma 1, oligodendroglioma 1, craniopharyngioma 1, retinoblastoma 1 rhabdomyosarcoma 1, scalp hemangioma 1, pineal mass 1, experimental	39.5	10/9	42.8 (*n* = 5)	29.7	margin, 12.9 maximum, 26.2	7.6	5, gr. I 2, gr. II 12, N/A	3.4	1 (5.3%) −1, optic neuropathy	6/24 (25%)

Kuhn et al., 2012 [15]	12	1, epidermoid carcinoma 2, astrocytoma 1, medulloblastoma 1, tinea capitis 1, lymphoma 1, scalp hemangioma 1, oligodendroglioma 1, neuroblastoma 1, ependymoma 1, pituitary tumor 1, pilocytic astrocytoma	53.1	7/5	52.9 (*n* = 3)	34.8	margin, 13.6	1.7	2, gr. I 4, gr. II 6, N/A	2.5	2 (16.7%) −1, CN V, VII, and VIII neuropathy −1, left leg weakness and numbness	2/24 (8.3%)

*Age at diagnosis of radiation-induced meningioma.

ALL: acute lymphoblastic leukemia; N/A: not available; NOS: not otherwise specified; RT: radiation therapy.

**Table 2:** Select series of surgically treated radiation-induced meningiomas.

Author and year	*N*	Original diagnosis for RT	Mean age (yrs.)∗	F/M	Mean RT dose (Gy)	Mean latency (yrs.)	Histology	WHO grade	Mean follow-up (yrs.)	Recurrence
Harrison et al., 1991 [5]	7	2, tinea capitis 1, presumed sarcoma 1. prepubertal gigantism 1, thalamic glioma 1, keloid 1, astrocytoma	53.4	5/2	—	42.7	3, atypical 2, meningothelial 1, transitional 1, mixed (meningothelial, transitional, fibroblastic)	—	—	3 (42.9%)

Salvati et al., 1996 [29]	10	4, ALL 2, medulloblastoma 2, adenoma 1, carcinoma 1, astrocytoma	30.4	7/3	39.8	16.9	4, atypical 4, fibroblastic 1, meningothelial 1, transitional	—	—	1 (10%)

Sadetzki et al., 2002 [30]	253^†^	253, tinea capitis	43.6	1.9 : 1	—	36.3	183, NOS 63, meningothelial 38, transitional 31, fibroblastic 4, angioblastic 2, psammomatous 1, malignant 6, mixed	—	9.8	18.2%

De Tommasi et al., 2005 [31]	6	6, tinea capitis	57.7	4/2	15	40.2	2, meningothelial 2, transitional 1, choroid 1 atypical	4, I 2, II	—	0

Banerjee et al., 2009 [32]	8	8, ALL	26.5	5/3	26.5	23	3, meningothelial 2, fibroblastic 2, transitional 1, atypical	7, I 1, II	—	2 (25%)

Godlewski et al., 2012 [33]	19	4, ALL 3, NHL 3, astrocytoma 2, ependymoma 1, pituitary adenoma 1, medulloblastoma 1, “brain tumor” 1, CNS lymphoma 1, leukemia 1, germinoma 1, ganglioneuroblastoma	35.7	12/7	31.9	25.9	—	17, I 1, II 1, N/A	5.8	4 (21.1%)

*Age at diagnosis of radiation-induced meningioma.

^†^220/253 patients were treated by surgical resection.

ALL: acute lymphoblastic leukemia; N/A: not available; NHL: non-Hodgkin's lymphoma; NOS: not otherwise specified; RT: radiation therapy.
